# The Effect of *Zataria multiflora* on Clinical, Pulmonary Function, and Oxidant Factors in Patients With Pulmonary Diseases: A Meta‐Analysis of Clinical Trials

**DOI:** 10.1002/hsr2.70772

**Published:** 2025-05-05

**Authors:** Amirali Aali, Erfan Taherifard, Melika Farshidianfar, Farshad Hadianfard, Fatemeh Alamdari, Omid Keshavarzian, Majid Nimrouzi, Seyed Taghi Heydari, Maryam Akbari

**Affiliations:** ^1^ Health Policy Research Center, Institute of Health Shiraz University of Medical Sciences Shiraz Iran; ^2^ Shiraz School for Medicine Shiraz University of Medical Sciences Shiraz Iran; ^3^ Research Center for Traditional Medicine and History of Medicine Shiraz University of Medical Sciences Shiraz Iran

**Keywords:** anti‐inflammatory agents, antioxidants, lung diseases, respiratory function tests, *Zataria multiflora* Boiss

## Abstract

**Background and Aims:**

In this study, we investigated the effects of *Z. multiflora* on clinical presentation, pulmonary function, and inflammatory and oxidative parameters in patients with pulmonary disorders.

**Methods:**

PubMed, Embase, Web of Science, Scopus, and Cochrane Library databases were searched to identify trials that assessed the effects of *Z. multiflora* in patients with different pulmonary diseases. A random‐effects model was then applied to estimate the weighted mean differences (WMDs) with 95% confidence intervals (CIs) of the outcomes considered in this meta‐analysis. Additional analyses were performed.

**Results:**

Eight articles were included in this meta‐analysis. *Z. multiflora* supplementation was associated with a significant increase in forced vital capacity (15.17%, 95% CI: 11.01–19.33), forced expiratory volume in 1 s (9.22%, 95% CI: 1.73–16.70), peak expiratory flow (8.68%, 95% CI: 5.94–11.42), catalase (0.05 U/mL, 95% CI: 0.01–0.10), thiol (0.02 μmol/mL, 95% CI: 0.01–0.03), interleukin‐10 (0.38 pg/mL, 95% CI: 0.34–0.42) and interferon‐gamma (0.74 pg/mL, 95% CI: 0.56–0.91). Besides, there were significant decreases in chest wheeze (−1.24, 95% CI: −1.48 to 1.01), malondialdehyde (−2.63 nmol/mL, 95% CI: −3.86 to −1.39), and tumor necrosis factor‐alpha levels (−1.45 pg/mL, 95% CI: −2.01 to −0.89). However, there were no significant changes in mid‐maximum expiratory flow and superoxide dismutase levels.

**Conclusions:**

The current meta‐analysis underscores the effects of Z. multiflora in improving the clinical manifestations of patients with pulmonary disease, as well as its anti‐inflammatory, immunomodulatory, and antioxidant effects. However, more trials with larger samples and longer observation durations are needed to strengthen the evidence for widespread supplementation of *Z. multiflora*.

**Abbreviations**
CAT: catalase
CI: confidence interval
COPD: chronic pulmonary lung disease
FEV1: forced expiratory volume in 1 s
FVC: forced vital capacity
IFN‐γ: interferon‐gamma
IL‐10: interleukin‐10
MDA: malondialdehyde
MMEF: mid‐maximum expiratory flow
NF‐κB: nuclear factor‐kappa B
PEF: peak expiratory flow
PFT: pulmonary function test
PRISMA: preferred reporting items for systematic reviews and meta‐analyses
RCT: randomized clinical trial
RoB: risk of bias
SD: standard deviation
SE: standard error
SOD: superoxide dismutase
TNF‐α: tumor necrosis factor‐alpha
WMD: weighted mean difference

## 1 Introduction

*Zataria multiflora* Boiss., *Z. multiflora* is an aromatic herbal plant belonging to the Lamiaceae family [1]. This herbal plant is mainly found in southwestern Asia and the Middle East, particularly Iran, Afghanistan, and Pakistan, where it has been widely used for food purposes because of its unique taste and aroma [2, 3]. The leaves and flowers of *Z. multiflora*, generally known as “Avishan‐e‐Shirazi” in Persian, were also collected for their therapeutic properties. The main compounds found in *Z. multiflora* are two phenolic monoterpene isomers, carvacrol and thymol, followed by p‐cymene, linalool, borneol, and γ‐terpinene [4, 5]. These compounds have potent anti‐inflammatory, antioxidative, antineoplastic, analgesic, antimicrobial, and antispasmodic properties [6, 7, 8, 9, 10, 11].

*Z. multiflora* has been frequently used in traditional medicine for the treatment of various diseases, such as pulmonary diseases. The potential of *Z. multiflora* in the treatment of pulmonary diseases has been demonstrated in several experimental and clinical trials [12]. Activation of beta‐2 adrenergic receptors, inhibition of histamine H1 and muscarinic receptors, prevention of alveolar wall destruction and emphysematous changes in lung parenchyma, and reduction of tracheal responsiveness, inflammatory markers, and oxidative stress are among the mechanisms exerted by *Z. multiflora* [13, 14, 15, 16, 17]. These beneficial effects have also been reported in several clinical studies conducted in patients with different pulmonary diseases [12]. A double‐blind randomized clinical trial (RCT) demonstrated that supplementation with *Z. multiflora* was associated with improvement in various symptoms, pulmonary function tests (PFTs), and oxidative parameters in patients with chronic obstructive pulmonary disease (COPD) [18]. In this study, it was shown that the C‐reactive protein level decreased significantly in patients receiving this herbal plant. Ghorani et al. conducted a recent study that demonstrated a notable reduction in respiratory symptoms, decreased usage of inhaled bronchodilator drugs, lower levels of total white blood cells (WBC) and neutrophils, and an improvement in the value of forced expiratory volume in 1 s (FEV1) in patients with COPD who received an extract derived from Z. multiflora. The evidence demonstrates the therapeutic effects of this herb on patients with COPD, potentially attributable to its anti‐inflammatory properties [19]. In addition to its beneficial medicinal effects, Z. multiflora has mild side effects that are often reported, including dizziness, skin irritation, nausea, vomiting, and stimulation of mucosal membranes [2].

The findings of these studies suggest that *Z. multiflora* may be a promising treatment option for pulmonary disorders; however, no consensus has been yet made on the effects that could be exerted by the use of *Z. multiflora* in patients with pulmonary diseases. In this study, we aimed to systematically review the literature to investigate the effects of *Z. multiflora* on the clinical presentation, PFTs, and different biochemical inflammatory and oxidative parameters in patients with pulmonary disorders.

## 2 Materials and Methods

This systematic review and meta‐analysis was conducted based on the preferred reporting items for systematic reviews and meta‐analyses (PRISMA) guidelines [20].

### 2.1 Search Strategy

Eligible clinical trials were searched systematically using the following electronic databases: PubMed, Scopus, Embase, Web of Science (ISI), and Cochrane Library before March 1, 2023. We also evaluated gray literature using the Google Scholar search engine and by communicating with experts and relevant research centers. In addition, we manually checked the reference lists of the relevant review studies and clinical trials to identify potential records.

In this study, we only retrieved trials that have investigated the effects of *Z. multiflora* on the clinical symptoms, PFTs, oxidant/antioxidant parameters, and cytokine levels by using the following MeSH and text words: patients (“lung disease” OR “lung disorder” OR “pulmonary disease” OR “pulmonary disorder” OR “chronic obstructive pulmonary disease” OR “COPD” OR “asthma”), intervention (“*Zataria multiflora* Boiss” OR” *Zataria multiflora*” OR “*Z. multiflora*”), and outcomes (“symptoms “OR “clinical symptoms “OR “pulmonary function tests “OR “oxidant/antioxidant parameters “OR “cytokine levels”). We limited our search to human trials and studies published in the English language.

### 2.2 Study Selection

Two independent researchers (A.A. and M.F.) screened titles and abstracts of all citations to remove irrelevant records. They then assessed the full texts of the remaining studies to determine whether they met the following criteria for inclusion: being an original human clinical trial that assessed the effects of *Z. multiflora* supplementation in patients with pulmonary diseases and reported sufficient data to calculate the mean changes and standard deviations (SDs) of changes in clinical symptoms, PFTs, oxidant/antioxidant parameters, and cytokine levels. However, we excluded studies that were case reports, case series, letters to editor studies, observational studies, and conference abstracts.

### 2.3 Data Extraction and Risk of Bias Assessment

A.A. and F.A. extracted information using an Excel spreadsheet and assessed the methodological quality of all eligible trials using the Cochrane Collaboration risk of bias tool. Any discrepancies were resolved by discussion with a third researcher (M.A).

The following data were extracted from eligible trials: (1) first author's name; (2) year of publication; (3) study location; (4) main demographic characteristics; (5) the total number of participants in each of the treatment and placebo groups; (6) study method; (7) types and doses of *Z. multiflora*; (8) duration of the study; and (9) the mean (SD) changes in the clinical symptoms (chest wheeze), PFTs (force vital capacity, FVC, peak expiratory flow, PEF, forced expiratory volume in 1 s, FEV1, and mid‐maximum expiratory flow, MMEF), oxidant/antioxidant system parameters (catalase, CAT, malondialdehyde, MDA, superoxide dismutase, SOD, and thiol), and cytokine levels (tumor necrosis factor‐alpha, TNF‐α, interleukin‐10, IL‐10, and interferon‐gamma, IFN‐γ) at the baseline of the studies and the end of treatment for each group. Notably, only outcomes examined in at least three studies were selected for the present meta‐analysis.

The Cochrane Collaboration risk of bias tool assesses the following domains: “randomization generation, allocation concealment, blinding of subjects and outcome assessment, incomplete outcome data, selective outcome reporting, and other sources of bias” [21].

### 2.4 Statistical Analysis

A random‐effects model [DerSimonian–Laird method] was applied using Stata v.11 (Stata Corp., College Station, TX, USA) to estimate weighted mean differences (WMDs) along with 95% confidence intervals (CIs) of the outcomes considered in this meta‐analysis. If trials did not report the mean (SD) change, the following formulas were used to calculate the mean changes and their corresponding SDs: [mean _post_ – mean _pre_] and [√([SD_pre_^2^ + SD_post_^2^] – [2 × R × SD_pre_ × SD_post_])], respectively [22]. A correlation coefficient of 0.5 was used when it was not reported in the studies [22]. To calculate the SD in trials that reported standard error (SE), the following formula was used: SE × √n, where *n* is the number of participants. Inter‐study heterogeneity across the trials was calculated using Cochran's Q test and the I‐square statistic (*p* < 0.1, *I* ^2^ > 50%) [23]. Additional analyses, such as subgroup analyses, were also performed based on the dosage of *Zataria multiflora* (≤ 5 mg/kg/day vs. > 5 mg/kg/day) and type of pulmonary diseases (COPD vs. asthma vs. lung diseases induced by exposure to sulfur mustard) to determine the source of heterogeneity. Sensitivity analyses using the leave‐one‐out method were conducted to assess the robustness of our findings by discarding each trial in turn. Egger's regression test was used to detect any potential evidence of publication bias in the analyses conducted for the outcomes. Statistical significance was set at *p* < 0.05.

## 3 Results

### 3.1 Search Results and Study Characteristics

**Figure 1:**
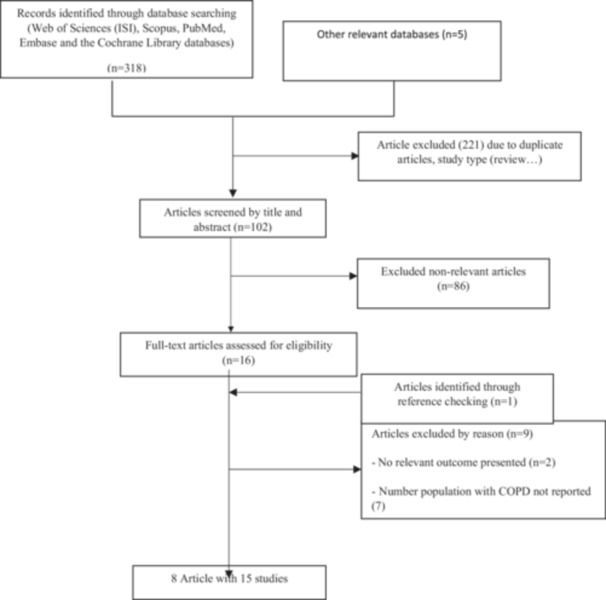
The PRISMA flowchart of the study identification and selection process.

A PRISMA flowchart explaining the process of identifying and selecting of studies in the literature search is presented in Figure 1. A total of 323 records were obtained through literature search. Of these, 221 were duplicates or irrelevant. After checking the titles and abstracts, the full texts of 16 papers were retrieved for a second round of screening according to our inclusion criteria. Eight articles (15 studies) were included in the current systematic review and meta‐analysis [18, 24, 25, 26, 27, 28].

All the included articles had a parallel design. The trials will be published between 2018 and 2022. Six trials investigated changes in chest wheeze, FVC, MDA, and thiol; seven assessed changes in PEF, FEV1, and MMEF; and four assessed changes in CAT, SOD, TNF‐α, IL‐10, and IFN‐γ levels. The main characteristics of the trials are summarized in Table 1.

**Table 1:** Main characteristics of the included trials assessing the effects of *Z. multiflora* in patients with pulmonary diseases.

Authors	Publication year	Country	Patients	Sample size (intervention vs. control)	Type of Study	Duration of follow‐up	Medication (control group)	Medication (Intervention group)	Measured outcomes
Ghorani et al. #1 (Alavinezhad et al., 2022)	2022	Iran	Moderate COPD patients	14/7	Randomized, double‐blind clinical trial	8 weeks	Placebo	3 mg/kg/day *Z. multiflora* extract	TNF‐α, FVC, PEF, FEV1,
Ghorani et al. #2 (Boskabady et al., 2020)	2022	Iran	Moderate COPD patients	14/6	Randomized, double‐blind clinical trial	8 weeks	Placebo	6 mg/kg/day *Z. multiflora* extract	TNF‐α, FVC, PEF, FEV1,
Alavinezhad et al. #1 (Alavinezhad et al., 2022)	2022	Iran	Asthmatic patients with moderate to severe asthma	12/6	Randomized, double‐blind clinical trial	8 weeks	Placebo	5 mg/kg/day *Z. multiflora* extract	IL‐10, FVC, PEF, MMEF,
Alavinezhad et al. #2 (Alavinezhad et al., 2022)	2022	Iran	Asthmatic patients with moderate to severe asthma	12/6	Randomized, double‐blind clinical trial	8 weeks	Placebo	10 mg/kg/day *Z. multiflora* extract	IL‐10, FVC, PEF, MMEF,
Alavinezhad et al. #1 (Boskabady et al., 2020)	2020	Iran	Moderate asthmatic patients	12/6	Randomized, double‐blind clinical trial	8 weeks	Placebo	5 mg/kg/day *Z. multiflora* extract	CAT, MDA, IFN‐γ, SOD, thiol, FEV1, chest wheeze
Alavinezhad et al. #2 (Boskabady et al., 2020)	2020	Iran	Moderate asthmatic patients	12/6	Randomized, double‐blind clinical trial	8 weeks	Placebo	10 mg/kg/day *Z. multiflora* extract	CAT, MDA, IFN‐γ, SOD, thiol, FEV1, chest wheeze
Boskabady et al. (Khazdair et al., 2020)	2020	Iran	Asthmatic patients	18/12	Clinical trial	180 min	Theophylline	The extract of *Z. multiflora* (20 mg/kg)	PEF, FEV1, MMEF,
Ghorani et al. #1 (Ghorani et al., 2020)	2020	Iran	Mild to moderate COPD	13/6	Randomized double‐blind clinical trial	8 weeks	Placebo	3 mg/kg/day *Z. multiflora* extract	CAT, MDA, SOD, thiol, FEV1, MMEF, chest wheeze
Ghorani et al. #2 (Ghorani et al., 2020)	2020	Iran	Mild to moderate COPD	16/7	Randomized double‐blind clinical trial	8 weeks	Placebo	6 mg/kg/day *Z. multiflora* extract	CAT, MDA, SOD, thiol, FEV1, MMEF, chest wheeze
Khazdair et al. #1 (Khazdair et al., 2020)	2020	Iran	Sulfur mustard‐induced lung disorders patients	11/6	Randomized, double‐blind clinical trial	8 weeks	Placebo	5 mg/kg/day *Z. multiflora* syrup	IL‐10, IFN‐γ, MMEF,
Khazdair et al. #2 (Khazdair et al., 2020)	2020	Iran	Sulfur mustard‐induced lung disorders patients	11/6	Randomized, double‐blind clinical trial	8 weeks	Placebo	10 mg/kg/day *Z. multiflora* syrup	IL‐10, IFN‐γ, MMEF,
Khazdair et al. #1 (Khazdair et al., 2020)	2020	Iran	Veterans exposed to sulfur mustard	11/6	Randomized, double‐blind clinical trial	8 weeks	Placebo	5 mg/kg/day *Z. multiflora* extract	TNF‐α, chest wheeze
Khazdair et al. #2 (Khazdair et al., 2020)	2020	Iran	Veterans exposed to sulfur mustard	11/6	Randomized, double‐blind clinical trial	8 weeks	Placebo	10 mg/kg/day *Z. multiflora* extract	TNF‐α, chest wheeze
Khazdair et al. #1 (Khazdair et al., 2018)	2018	Iran	Sulfur mustard exposed veterans	11/6	Randomized, double‐blind clinical trial	8 weeks	Placebo	5 mg/kg/day *Z. multiflora* extract	MDA, thiol, FVC, PEF,
Khazdair et al. #2 (Khazdair et al., 2018)	2018	Iran	Sulfur mustard exposed veterans	11/7	Randomized, double‐blind clinical trial	8 weeks	Placebo	10 mg/kg/day *Z. multiflora* extract	MDA, thiol, FVC, PEF,

Abbreviations: CAT, catalase; COPD, chronic obstructive pulmonary disease; FEV1, forced expiratory volume in 1 s; FVC, forced vital capacity; IFN‐γ, interferon‐gamma; IL‐10, interleukin 10; MDA, malondialdehyde; MMEF, mid‐maximum expiratory flow; PEF, peak expiratory flow; SOD, superoxide dismutase; TNF‐α and tumor necrosis factor‐alpha.

### 3.2 The Effects of *Z. multiflora* on Clinical Manifestation

The effect of *Z. multiflora* on chest wheezing in patients with pulmonary disease is shown in Figure 2A. Using the random‐effects model, the meta‐analysis indicated a significant decrease in the WMD of chest wheeze in patients receiving *Z. multiflora* compared to patients in the control group [No. trials = 6; WMD = −1.24, 95% CI: −1.48, −1.01; *I* ^2^ = 90.63%, *p* < 0.001].

Figure 2(A–L) The effects of Z. multiflora on (A) chest wheeze, (B) FVC, (C) PEF, (D) FEV1, (E) MMEF, (F) CAT, (G) thiol, (H) MDA, (I) SOD, (J) TNF‐α, (K) IL‐10, (L) IFN‐γ in patients with pulmonary diseases.

**Figure:**
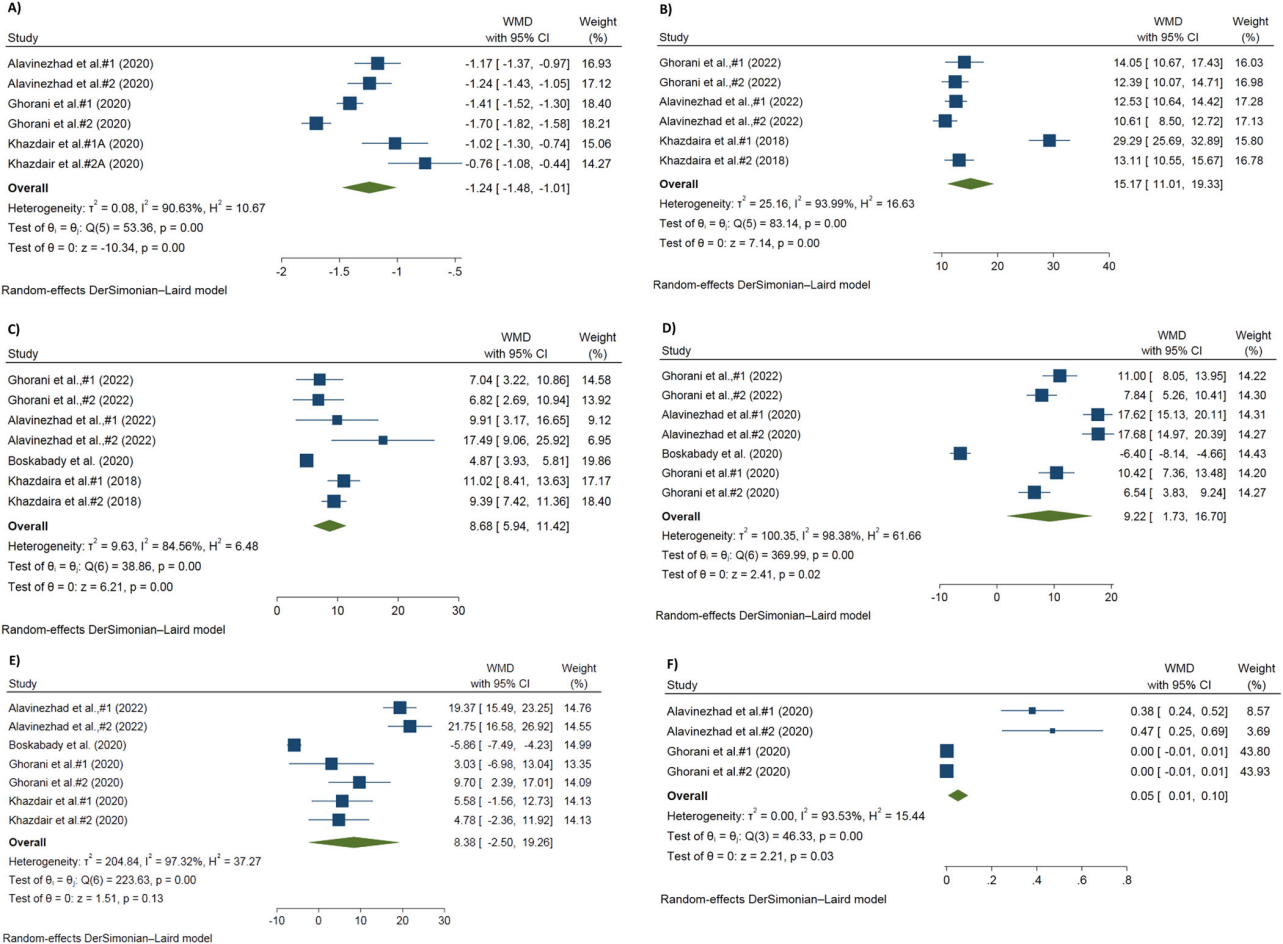

**Figure:**
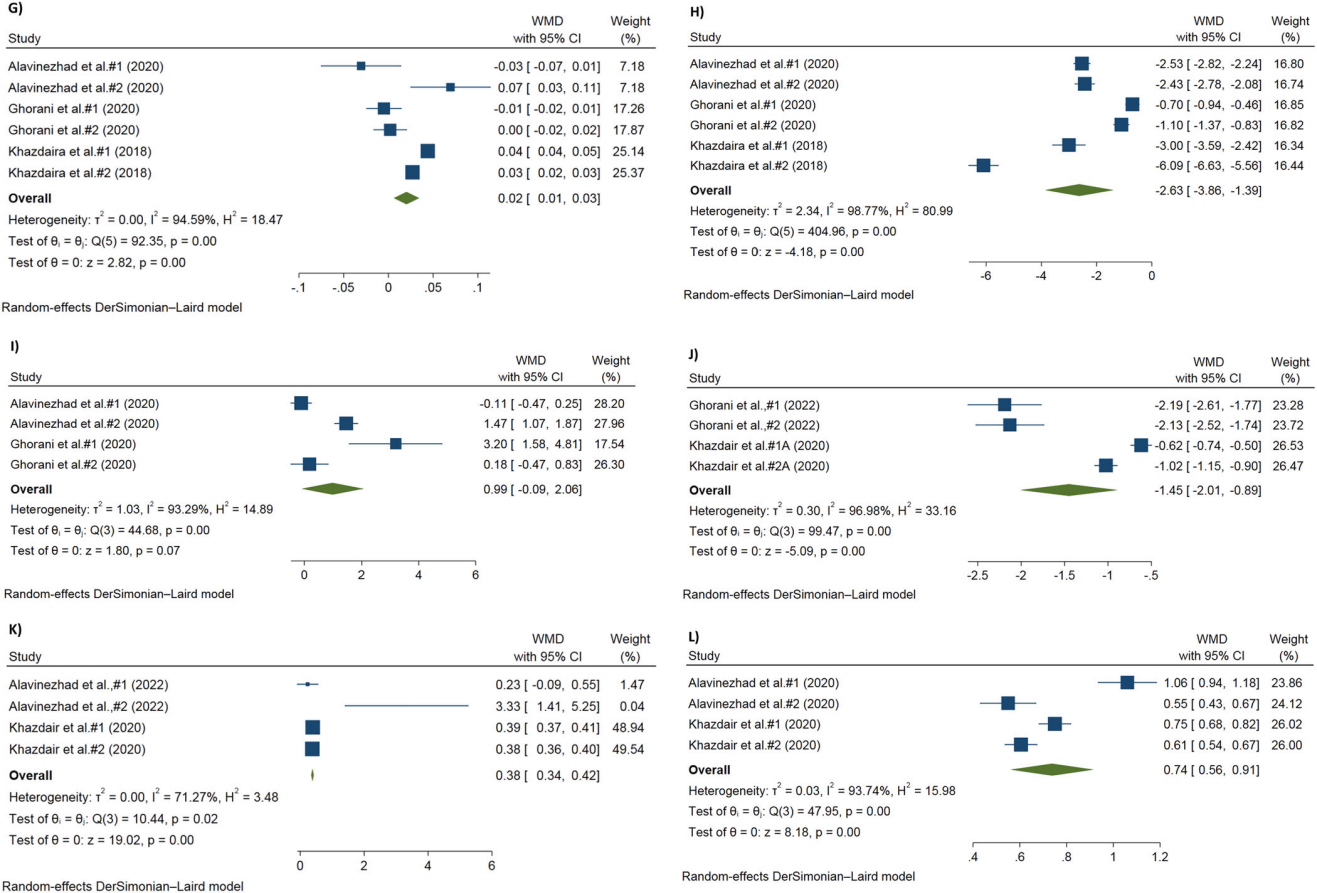

### 3.3 The Effects of *Z. multiflora* on PFTs

Figure 2B–E show the results of the quantitative analyses of PFTs. Significant increases in FVC [No. trials = 6; WMD = 15.17%, 95% CI: 11.01, 19.33; *I* ^2^ = 93.99%, *p* < 0.001], PEF [No. trials = 7; WMD = 8.68%, 95% CI: 5.94, 11.42; *I* ^2^ = 84.56%, *p* < 0.001], and FEV1 [No. trials= 7; WMD = 9.22%, 95% CI: 1.73, 16.70; *I* ^2^ = 98.38%, *p* < 0.001] were observed in the treatment group compared with those in the control group. However, the analysis showed no significant change in the MMEF [No. trials = 7; WMD = 8.38%, 95% CI: −2.50, 19.26; *I* ^2^ = 97.32%, *p* < 0.001] between these two groups.

### 3.4 The Effects of *Z. multiflora* on Oxidant/Antioxidant Parameters

Forest plots showing the effects of *Z. multiflora* supplementation on oxidant/antioxidant parameters are presented in Figure 2F–I. The pooled findings using the random‐effects model showed that treatment with *Z. multiflora* significantly increased the levels of CAT [No. trials = 4; WMD = 0.05 U/mL, 95% CI: 0.01, 0.10; *I* ^2^ = 93.53%, *p* < 0.001] and thiol [No. Trials = 6; WMD = 0.02 μmol/mL, 95% CI: 0.01, 0.03; *I* ^2^ = 94.59%, *p* < 0.001] and significantly decreased MDA levels [No. trials = 6; WMD = −2.63 nmol/mL, 95% CI: −3.86, −1.39; *I* ^2^ = 98.77%, *p* < 0.001]. However, there were no significant changes in the levels of SOD [No. trials = 4; WMD = 0.99 U/mL, 95% CI: −0.09, 2.06; *I* ^2^ = 93.29%, *p* < 0.001] following administration of *Z. multiflora*.

### 3.5 The Effects of *Z. multiflora* on Cytokine Levels

The pooled results for changes in cytokine levels indicated that *Z. multiflora* significantly decreased the level of TNF‐α [No. trials = 4; WMD = −1.45 pg/mL, 95% CI: −2.01, −0.89; *I* ^2^ = 96.98%, *p* < 0.001], and significantly increased levels of both IL‐10 [No. trials = 4; WMD = 0.38 pg/mL, 95% CI: 0.34, 0.42; *I* ^2^ = 71.27%, *p* = 0.02] and IFN‐γ [No. trials = 4; WMD = 0.74 pg/mL, 95% CI: 0.56, 0.91; *I* ^2^ = 93.74%, *p* < 0.001] (Figure 2J–L).

### 3.6 Subgroup Analyses

As shown in Table 2, the effects of *Z. multiflora* on patients with pulmonary disease were analyzed using a set of subgroups. We found that some outcomes, including chest wheeze, FVC, FEF, MDA, SOD, and IFN‐γ, did not change in the subgroup analyses based on the type of disease and dosage of treatment.

**Table 2:** The results of subgroup analyses of the effects of *Z. multiflora* in patients with pulmonary diseases.

Variables	Subgroup	No. study	WMD (95% CI)	Heterogeneity (*I* ^2^, *p*)
**Clinical symptom**				
**Chest wheeze**		6	−1.24 (−1.48, −1.01)	90.63%, < 0.001
** Patients**	Asthma	2	−1.20 (−1.34, −1.07)	0.00%, 0.612
	COPD	2	−1.55 (−1.83, −1.26)	91.43%, < 0.01
	Sulfur mustard exposed	2	−0.90 (−1.15, −0.64)	31.26%, 0.228
Dosage	≤ 5 mg/kg/day	3	−1.22 (−1.45, −0.99)	78.16%, 0.010
	> 5 mg/kg/day	3	−1.25 (−1.74, −0.76)	94.79%, < 0.001
**Pulmonary Function Tests**				
**FVC**		6	15.17 (11.01, 19.33)	93.99%, < 0.001
Patients	Asthma	2	11.62 (9.75, 13.50)	43.23%, 0.184
	COPD	2	12.92 (11.01, 14.83)	0.00%, 0.425
	Sulfur mustard exposed	2	21.15 (5.29, 37.01)	98.06%, < 0.001
Dosage	≤ 5 mg/kg/day	3	18.54 (8.88, 28.21)	96.99%, < 0.001
	> 5 mg/kg/day	3	11.90 (10.42, 13.39)	18.95%, 0.291
**PEF**		7	8.68 (5.94, 11.42)	84.56%, < 0.001
Patients	Asthma	3	9.80 (2.64, 16.96)	80.90%, < 0.01
	COPD	2	6.93 (4.13, 9.73)	0.00%, 0.939
	Sulfur mustard exposed	2	9.98 (8.40, 11.55)	00.0%, 0.330
Dosage	≤ 5 mg/kg/day	3	9.53 (6.85, 12.20)	29.67%, 0.241
	> 5 mg/kg/day	4	8.25 (4.60, 11.90)	87.57%, < 0.001
**FEV1**		7	9.22 (1.73, 16.70)	98.38%, < 0.001
Patients	Asthma	3	9.61 (−7.79, 27.02)	99.43%, < 0.001
	COPD	4	8.84 (6.78, 10.91)	53.36%, 0.092
	Sulfur mustard exposed	—	—	—
Dosage	≤ 5 mg/kg/day	3	13.07 (8.29, 17.85)	88.33%, < 0.001
	> 5 mg/kg/day	4	6.38 (4.37, 17.13)	98.78%, < 0.001
**MMEF**		7	8.38 (−2.50, 19.26)	97.32%, < 0.001
Patients	Asthma	3	11.66 (−8.62, 31.95)	99.06%, < 0.001
	COPD	2	7.27 (0.98, 13.56)	10.20%, 0.291
	Sulfur mustard exposed	2	5.18 (0.13, 10.23)	0.00%, 0.877
Dosage	≤ 5 mg/kg/day	3	9.91 (−1.43, 21.26)	88.25%, < 0.001
	> 5 mg/kg/day	4	7.50 (−7.27, 222.27)	97.40%, < 0.001
**Oxidant/Antioxidant parameters**				
CAT (U/mL)		4	0.05 (0.01, 0.10)	93.53%, < 0.001
Patients	Asthma	2	0.40 (0.28, 0.52)	0.00%, 0.501
	COPD	2	0.00 (−0.01, 0.01)	0.00%, 0.892
	Sulfur mustard exposed	—	—	—
Dosage	≤ 5 mg/kg/day	2	0.18 (−0.18, 0.55)	96.57%, < 0.001
	> 5 mg/kg/day	2	0.22 (−0.23, 0.68)	94.15%, < 0.001
MDA (nmol/mL)		6	−2.63 (−3.86, −1.39)	98.77%, < 0.001
Patients	Asthma	2	−2.48 (−2.71, −2.26)	0.00%, 0.665
	COPD	2	−0.89 (−1.28, −0.50)	78.20%, 0.032
	Sulfur mustard exposed	2	−4.55 (−7.58, −1.51)	98.30%, < 0.001
Dosage	≤ 5 mg/kg/day	3	−2.06 (−3.52, −0.60)	98.24%, < 0.001
	> 5 mg/kg/day	3	−3.19 (−5.65, −0.74)	99.26%, < 0.001
SOD (U/mL)		4	0.99 (−0.09, 2.06)	93.29%, < 0.001
Patients	Asthma	2	0.67 (−0.87, 2.22)	96.99%, < 0.001
	COPD	2	1.59 (−1.35, 4.54)	91.28%, < 0.001
	Sulfur mustard exposed	—	—	—
Dosage	≤ 5 mg/kg/day	2	1.44 (−1.79, 4.68)	93.47%, < 0.001
	> 5 mg/kg/day	2	0.85 (−0.40, 2.11)	90.79%, < 0.001
Thiol (μmol/mL)		6	0.02 (0.01, 0.03)	94.59%, < 0.001
Patients	Asthma	2	0.019 (−0.079, 0.117)	89.75%, < 0.001
	COPD	2	−0.001 (−0.015, 0.012)	0.00%, 0.604
	Sulfur mustard exposed	2	0.035 (0.019, 0.052)	98.26%, < 0.001
Dosage	≤ 5 mg/kg/day	3	0.006 (−0.038, 0.051)	94.18%, < 0.001
	> 5 mg/kg/day	3	0.025 (0.001, 0.050)	81.28%, 0.005
**Cytokine Levels**				
TNF‐α (pg/mL)		4	−1.45 (−2.01, −0.89)	96.98%, < 0.001
Patients	Asthma	—	—	—
	COPD	2	−2.15 (−2.44, −1.87)	0.00%, 0.838
	Sulfur mustard exposed	2	−0.82 (−1.21, −0.42)	95.23%, < 0.001
Dosage	≤ 5 mg/kg/day	2	−1.39 (−2.93, 0.15)	97.98%, < 0.001
	> 5 mg/kg/day	2	−1.56 (−2.64, −0.47)	96.40%, < 0.001
IL‐10 (pg/mL)		4	0.38 (0.34, 0.42)	71.27%, 0.020
Patients	Asthma	2	1.63 (−1.39, 4.65)	89.74%, 0.002
	COPD	—	—	—
	Sulfur mustard exposed	2	0.38 (0.37, 0.0.40)	0.00%, 0.475
Dosage	≤ 5 mg/kg/day	2	0.39 (0.37, 0.0.41)	0.00%, 0.332
	> 5 mg/kg/day	2	1.69 (1.18, 4.56)	88.98%, 0.003
IFN‐γ (pg/mL)		4	0.74 (0.56, 0.91)	93.74%, < 0.001
Patients	Asthma	2	0.80 (0.30, 1.30)	97.05%, < 0.001
	COPD	—	—	—
	Sulfur mustard exposed	2	0.67 (0.53, 0.82)	88.61%, 0.003
Dosage	≤ 5 mg/kg/day	2	0.90 (0.59, 1.20)	94.58%, < 0.001
	> 5 mg/kg/day	2	0.59 (0.53, 0.65)	0.00%, 0.430

Abbreviations: CAT, catalase; COPD, chronic obstructive pulmonary disease; FEV1, forced expiratory volume in 1 s; FVC, forced vital capacity; IFN‐γ, interferon‐gamma; IL‐10, interleukin 10; MDA, malondialdehyde; MMEF, mid‐maximum expiratory flow; PEF, peak expiratory flow; SOD, superoxide dismutase; TNF‐α, tumor necrosis factor‐alpha.

In subgroup analyses by patient type, changes in FEV1 in asthmatic patients (WMD = 9.61%, 95% CI: −7.79, 27.02), CAT levels in COPD patients (WMD = 0.00 U/mL, 95% CI: −0.01, 0.01), thiol levels in patients with asthma (WMD = 0.019 μmol/ml, 95% CI: −0.079, 0.117) and COPD (WMD = −0.001 μmol/mL, 95% CI: −0.015, 0.012), and IL‐10 levels in asthmatic patients (WMD = 1.63 pg/mL, 95% CI: −1.39, 4.65) were significantly altered. We found that in patients with COPD (WMD = 7.27%, 95% CI: 0.98, 13.56) and sulfur mustard‐exposed lung disease (WMD = 5.18%, 95% CI: 0.13, 10.23), *Z. multiflora* had a considerable effect on increasing the MMEF.

The levels of CAT (WMD = 0.18 U/mL, 95% CI: −0.18, 0.55), thiol (WMD = 0.006 μmol/ml, 95% CI: −0.038, 0.051), and TNF‐α (WMD = −1.39 pg/mL, 95% CI: −2.93, 0.15) were not significantly affected when subgroup analysis was conducted based on the dosage of the treatment (≤ 5 mg/kg/day).

### 3.7 Sensitivity Analysis

The sensitivity analyses indicated no significant changes between the pre‐ and post‐sensitivity analyses of the effect sizes after removing one by one of the included trials for changes in chest wheezing, FVC, PEF, CAT, MDA, TNF‐α, IL‐10, and IFN‐γ.

However, we found a significant change in the pooled WMD of FEV1, MMEF, thiol, and SOD in *Z. multiflora* compared to the control group after excluding Alavinezhad et al. #2 (2020) study [25] (WMD = 7.80, 95% CI: −0.07, 15.68), Boskabady et al. (2020) study [26] (WMD = 11.30, 95% CI: 4.66, 17.93), Khazdaira et al. (2018) study [27] (WMD = 0.016, 95% CI: −0.014, 0.046), and Alavinezhad et al. #1 (2020) study [25] (WMD = 1.41, 95% CI: 0.17, 2.64), respectively (Supporting Figure 1A–L).

### 3.8 Publication Bias and Risk of Bias Assessment

**Figure 3:**
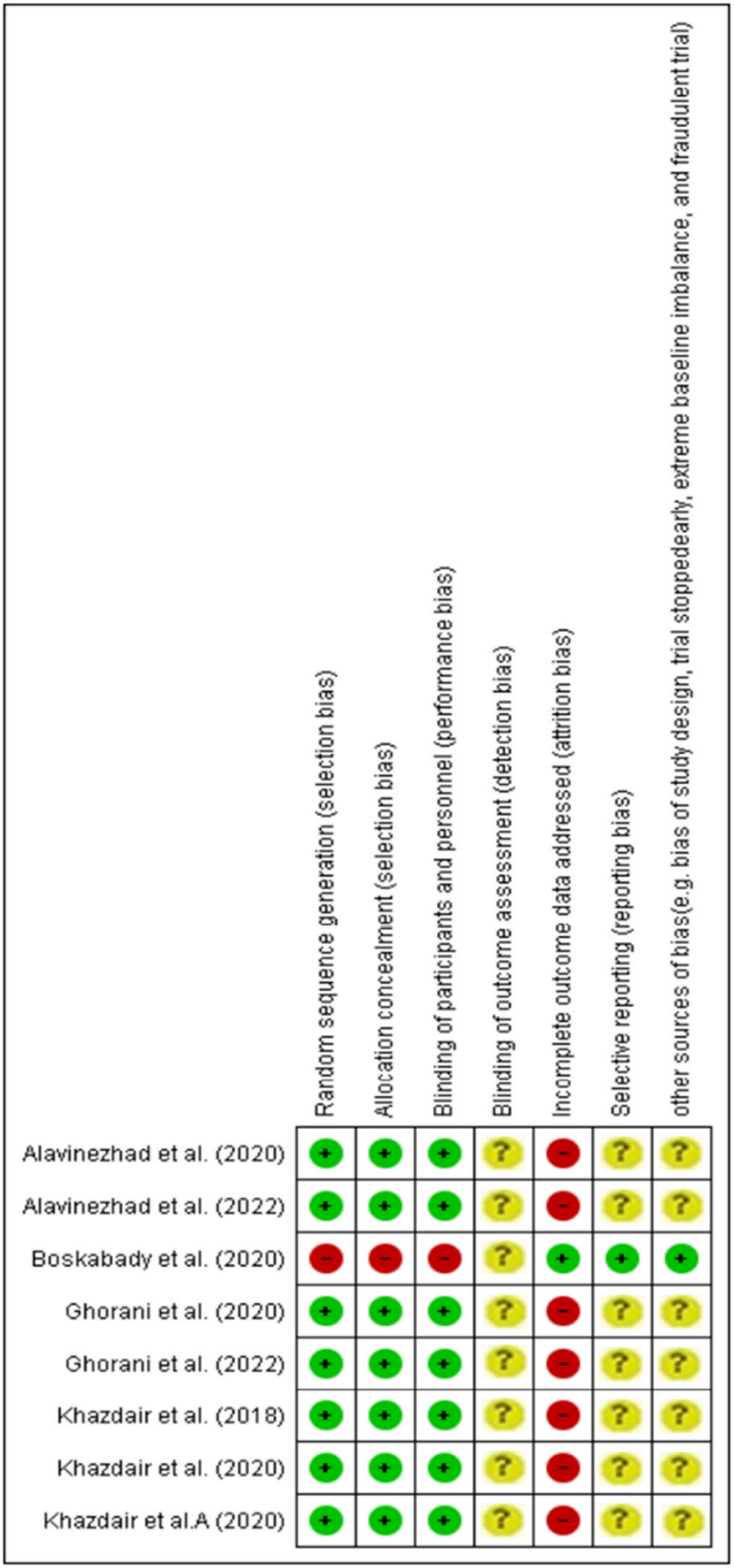
Risk of bias summary about each risk of bias item for the included trials.

Due to the few trials included for the assessment of the evidence of potential publication bias, we were not able to perform publication bias analyses. Figure 3 shows the results of (RoB) assessment of the included articles.

## 4 Discussion

To the best of our knowledge, the current study is the first meta‐analysis to investigate the effects of *Z. multiflora* in patients with pulmonary diseases. Eight articles were eligible for inclusion in the quantitative analyses. We found that supplementation with *Z. multiflora* was associated with improvement in chest wheezing and lung function in patients with pulmonary diseases. In addition, it was shown that *Z. multiflora* provided antioxidant properties with an increase in the levels of CAT and thiol and a decrease in the level of MDA. In addition, it exerts anti‐inflammatory and immunomodulatory effects in these patients, with a decrease in TNF‐α levels and an increase in IL‐10 and IFN‐γ levels.

Chronic airway and lung tissue inflammation and oxidative stress cause narrowing and respiratory damage in pulmonary diseases [29, 30, 31]. TNF‐α contributes to the initiation and progression of inflammation, oxidative stress, and cytotoxic cellular injuries in lung diseases [32]. This cytokine and its downstream pro‐inflammatory cytokines, IL‐1 and IL‐6, disrupt the redox balance between the oxidant and antioxidant systems in the airways and lung parenchyma by increasing the production of reactive oxygen species and decreasing the production of antioxidant scavengers and enzymes. Conversely, IL‐10, an anti‐inflammatory cytokine, is reduced in patients with pulmonary diseases and is negatively correlated with disease severity and progression [33, 34, 35]. All of these cytokines, TNF‐α, IL‐10, and IFN‐γ, are targets for treating different pulmonary diseases [36, 37]; for example, administration of recombinant human IFN‐γ results in favorable respiratory outcomes in patients exposed to sulfur mustard [38, 39]. This study found that *Z. multiflora* significantly reduced TNF‐α levels and increased IL‐10 and IFN‐γ levels, supporting previous studies on its effects on inflammatory cytokines [6, 40, 41, 42]. The analysis revealed high heterogeneity among the studies and methodological biases, which impacted methodological quality. Performing a subgroup analysis by adjusting the dosage helped decrease variability and highlighted the influence of the intervention dosage. This underscores the importance of considering the intervention dosage. For instance, a recent study conducted by Amin et al. investigated the impact of different doses of *Z. multiflora*, both alone and in combination with pioglitazone. The study found that treatment with ZME (200 and 400 mg/kg) altered the serum levels of nitrite, IL‐17, TNF‐α, IL‐10, and interferon‐gamma in rats with systemic inflammation induced by paraquat [43]. Hence, it is imperative to carry out more comprehensive research to elucidate the observed results.

In addition to the beneficial changes in the biochemical aspects of patients with different pulmonary diseases, including oxidant/antioxidant markers and cytokine levels, statistical analyses showed that supplementation with *Z. multiflora* resulted in significant changes in clinical aspects. Included studies reported that treatment with this herbal plant decreased the severity of clinical symptoms, such as cough, chest tightness, chest wheezing, and wheezing during exercise and during the night; however, we were only able to perform an analysis on the severity of chest wheeze, which was indicated to decrease significantly following the use of *Z. multiflora*. Additionally, the findings of our research demonstrated a substantial increase in FVC, PEF, and FEV1 in the treatment group compared to the control group and increased MMEF in patients with COPD and sulfur mustard‐exposed lung disease, as shown by the subgroup analysis. Expiratory airflow limitation and decreases in FVC, FEV1, and PEF are hallmarks of obstructive lung diseases, and mitigation of this decrease is important in the management of patients [44, 45]. Several mechanisms may be responsible for the beneficial effects of *Z. multiflora* extract. In addition to its anti‐inflammatory, immunomodulatory, and antioxidative effects, *Z. multiflora* can exert bronchodilatory properties. This herbal plant stimulates beta‐2 adrenergic receptors and, at the same time, inhibits both histamine H1 receptors and muscarinic receptors, resulting in bronchodilation [13, 14, 15]. In a trial conducted by Boskabady et al., the effects of *Z. multiflora* extracts on PFTs in patients with asthma were compared to those of theophylline, a well‐established bronchodilator currently used in the management of obstructive lung diseases [25]. This study showed that the bronchodilatory effect of *Z. multiflora* was not only non‐inferior to that of theophylline, but was also sustained longer. However, this study was performed in only 18 patients, and only a single dose was administered. Therefore, further research is needed to fully elucidate and compare the bronchodilatory effect of *Z. multiflora* with that of frequently used bronchodilators in these patients.

In the lungs, reactive oxygen species (ROS)‐mediated oxidative stress can cause chronic inflammation, cellular senescence, poor autophagy, low DNA repair, autoimmunity, increased mucus production, and inefficient anti‐inflammatory response to corticosteroids. This deviation from normal cell function can explain the pathogenic features of pulmonary diseases, possibly accelerating disease development and comorbidities [46, 47]. These findings suggest that antioxidants can be used to control disease progression. Multiple studies have shown that Z. multiflora and its components are antioxidants and free radical scavengers, suggesting that phenolic compounds are the main antioxidants in Z. multiflora [48]. Some studies have found that Z. multiflora essential oil and methanolic extract have antioxidant activity by inhibiting free radicals using the DPPH and ammonium thiocyanate systems [49, 50]. Furthermore, it was demonstrated that carvacrol and essential oil of Z. multiflora inhibited arachidonic pathways and NO production in macrophages stimulated by LPS [51, 52].

Accounting the pooled findings of our study, administration of Z. multiflora resulted in a significant increase in CAT and thiol levels and a decrease in MDA levels. However, there were no significant changes in SOD levels after the administration of Z. multiflora. Furthermore, the effects of Z. multiflora on CAT and MDA levels in patients with asthma remained significant, with a decrease in heterogeneity following subgroup analysis. Consistent with our findings, prior studies have demonstrated that Z. multiflora and carvacrol mitigate oxidative stress [53, 54, 55, 56]. This finding may encourage future research with a larger sample size.

In addition to the properties found in this meta‐analysis, *Z. multiflora* supplementation may be beneficial in patients with chronic pulmonary diseases. Studies have reported that *Z. multiflora* and its main compounds, carvacrol and thymol, exert antimicrobial effects [12, 57]. Therefore, it may be beneficial in the prevention of exacerbations in these patients, which is among the most important goals of their management [58, 59]. In addition, it seems that *Z. multiflora* can prevent the progression of pulmonary diseases. In a multi‐arm experimental study conducted on Guinea Pigs, animals were exposed to cigarette smoke to induce COPD [16]. After completion of the study, lung tissue sections were obtained from the animals and analyzed for the extent of parenchymal destruction and emphysematous formations. The findings of this study demonstrated that the animals treated with *Z. multiflora* or carvacrol extracts showed a significant decrease in the severity of emphysematous changes in the lung tissue, suggesting a preventive effect of these agents.

This study has several limitations. Eight clinical trials (15 studies) were included in this study; however, for most of the outcomes, there were few eligible studies, and the overall number of participants was low. Second, substantial rates of inter‐study heterogeneity were observed in quantitative analyses. To identify sources of heterogeneity, we conducted subgroup analyses, and through these analyses, we were able to identify factors that contributed to the heterogeneity of some of the outcomes. Third, due to the few eligible studies for the assessment of each outcome, potential publication bias was not investigated. Therefore, due to the limitations that we had in the conduction of this systematic review and meta‐analysis, its findings should be interpreted with caution and should be consolidated with further well‐powered studies.

## 5 Conclusions

Overall, this systematic review and meta‐analysis showed that Z. multiflora is associated with improvements in the clinical manifestations of patients with pulmonary diseases and can also decrease chest wheezing, improve lung function, provide antioxidant properties, and exert anti‐inflammatory and immunomodulatory effects in these patients. Given the abundance and affordability of this herbal plant in southwestern Asian countries, such as Iran, Afghanistan, and Pakistan, Z. multiflora may be a promising option for treating patients with obstructive lung diseases and chemical‐induced lung diseases. However, the study acknowledges limitations such as a small number of eligible studies, a low overall number of participants, high heterogeneity, and potential publication bias. However, for widespread supplementation of Z. multiflora, a stronger body of evidence with further trials, larger sample sizes, and longer observation durations is required.

## Author Contributions

**Amirali Aali:** data curation, investigation, methodology, validation, writing – original draft. **Erfan Taherifard:** conceptualization, methodology, software, project administration, writing – original draft, writing – review and editing. **Melika Farshidianfar:** investigation, methodology, validation, writing – original draft. **Farshad Hadianfard:** investigation, methodology, data curation, writing – original draft. **Fatemeh Alamdari:** investigation, methodology, data curation, writing – original draft. **Omid Keshavarzian:** investigation, methodology, validation, writing – original draft. **Majid Nimrouzi:** conceptualization, formal analysis, investigation, methodology, writing – review and editing, supervision. **Seyed Taghi Heydari:** conceptualization, formal analysis, investigation, methodology, writing – review and editing, supervision. **Maryam Akbari:** conceptualization, formal analysis, investigation, methodology, writing – review and editing, supervision.

## Ethics Statement

The authors have nothing to report.

## Conflicts of Interest

The authors declare no conflicts of interest.

## Transparency Statement

The lead author Maryam Akbari affirms that this manuscript is an honest, accurate, and transparent account of the study being reported; that no important aspects of the study have been omitted; and that any discrepancies from the study as planned (and, if relevant, registered) have been explained.

## Supporting information

Supporting Figure 1.

## Data Availability

The data set used and analyzed during the current study are available from the corresponding author upon reasonable request.
