# Supplementary material for: The Effect of Zataria multiflora on Clinical, Pulmonary Function, and Oxidant Factors in Patients With Pulmonary Diseases: A Meta‐Analysis of Clinical Trials
Source: Health Sci Rep. 2025 May 5;8(5):e70772. doi: 10.1002/hsr2.70772 (PMC12052514; doi:10.1002/hsr2.70772)
Supplement: Supplementary file 1 — Supporting Figure 1. [file HSR2-8-e70772-s001.docx]

**Supplementary Figure 1A-L.** Sensitivity analyses findings of effects of Z. multiflora in patients with pulmonary diseases

**Supplementary Figure 1A.** Chest wheeze

**Supplementary Figure 1B.** FVC

**Supplementary Figure 1C.** PEF

**Supplementary Figure 1C.** FEV1

**Supplementary Figure 1E.** MMEF

**Supplementary Figure 1F.** CAT

**Supplementary Figure 1G.** thiol

**Supplementary Figure 1H.** MDA

**Supplementary Figure 1I.** SOD

**Supplementary Figure 1J.** TNF-α

**Supplementary Figure 1K.** IL-10

**Supplementary Figure 1L.** IFN-γ
